# Revealing Distinctions in Genetic Diversity and Adaptive Evolution Between Two Varieties of *Camellia sinensis* by Whole-Genome Resequencing

**DOI:** 10.3389/fpls.2020.603819

**Published:** 2020-11-24

**Authors:** Yanlin An, Xiaozeng Mi, Shiqi Zhao, Rui Guo, Xiaobo Xia, Shengrui Liu, Chaoling Wei

**Affiliations:** State Key Laboratory of Tea Plant Biology and Utilization, Anhui Agricultural University, Hefei, China

**Keywords:** tea plant, genome-resequencing, genetic diversity, adaptive evolution, indel markers

## Abstract

*Camellia sinensis* var. *sinensis* (CSS) and *C. sinensis* var. *assamica* (CSA) are the two most economically important tea varieties. They have different characteristics and geographical distribution. Their genetic diversity and differentiation are unclear. Here, we identified 18,903,625 single nucleotide polymorphisms (SNPs) and 7,314,133 insertion–deletion mutations (indels) by whole-genome resequencing of 30 cultivated and three wild related species. Population structure and phylogenetic tree analyses divided the cultivated accessions into CSS and CSA containing 6,440,419 and 6,176,510 unique variations, respectively. The CSS subgroup possessed higher genetic diversity and was enriched for rare alleles. The CSA subgroup had more non-synonymous mutations and might have experienced a greater degree of balancing selection. The evolution rate (dN/dS) and KEGG enrichment indicated that genes involved in the synthesis and metabolism of flavor substances were positively selected in both CSS and CSA subpopulations. However, there are extensive genome differentiation regions (2959 bins and approximately 148 M in size) between the two subgroups. Compared with CSA (141 selected regions containing 124 genes), the CSS subgroup (830 selected regions containing 687 genes) displayed more selection regions potentially related to environmental adaptability. Fifty-three pairs of polymorphic indel markers were developed. Some markers were located in hormone-related genes with distinct alleles in the two cultivated subgroups. These identified variations and selected regions provide clues for the differentiation and adaptive evolution of tea varieties. The newly developed indel markers will be valuable in further genetic research on tea plants.

## Introduction

The tea plant (*Camellia sinensis* (L.) O. Kuntze) is a perennial evergreen woody plant (2*n* = 2*x* = 30) that originated in southwest China. The plant was first cultivated over 2,000 years ago (Xia et al., 2017; Wei et al., 2018). Tea is one of the most popular non-alcoholic drinks worldwide, reflecting its economic and cultural importance, as well as health benefits. *C. sinensis* var. *sinensis* (CSS) and *C. sinensis* var. *assamica* (CSA) are two main varieties of tea trees and are the most widely cultivated varieties globally. CSA are mainly cultivated in tropical regions, while CSS are introduced into most tropical and subtropical regions due to its stronger cold resistance. The distinct morphological and biochemical characteristics between CSS and CSA are mainly attributed to their different geographical distributions and growth environments (Hao et al., 2018; Li et al., 2019). For example, CSA is generally rapidly growing with a larger leaf area compared with CSS. Significant differences in the contents of flavonoids, caffeine, and theanine have been demonstrated between the two varieties (Wei et al., 2018; Zhu et al., 2019). Tea plants are self-incompatible, which promotes relatively highly heterozygous progenies and may produce rich genetic variations. Therefore, revealing the interspecific differences and genetic diversity at the whole-genome level is important for the understanding of the genetic mechanism of tea plant growth and development.

With the loss and acquisition of genes, almost all cultivated crops have undergone different degrees of domestication and improvement to adapt to the environment and meet human needs. Different domestication and improvement purposes will result in different genome characteristics. With the rapid development of sequencing technology, whole-genome sequencing and large-scale resequencing can be applied to various plant species. Sequence alignment can detect many sites of variation in genetic and intergenic regions. This includes some non-synonymous mutations located in the coding or regulatory regions, which have become important resources for studying gene functional and genetic differentiation. Whole-genome resequencing has been widely applied in various plant species, such as the detection of variation sites in cotton (Shen et al., 2017; Wang et al., 2017; Du et al., 2018), genome association analysis in rice (Huang et al., 2013; Wang et al., 2016), origin and evolutionary analysis in pears (Wu et al., 2018), and quantitative trait locus (QTL) mapping of important agronomic traits in melo (Hu et al., 2017). A study reported that CSA and CSS may have diverged 22,000 years ago and were subsequently domesticated independently (Meegahakumbura et al., 2016). This may have resulted in different tree height, leaf area, resistance, and contents of quality component between the two subgroups. However, due to insufficient reliable evidence, the regions of divergence between CSS and CSA in the genome have not been elucidated.

Due to the high heterozygosity (2.7%) and large genome size (approximately 2.94 Gb), it is very difficult to explore the genetic differentiation of tea plants on the whole-genome scale (Xia et al., 2020b). With the determinations of the sequences of the “Yunkang 10” and “Shuchazao” genomes (Xia et al., 2017; Wei et al., 2018), it has become possible to study the domestication and improvement of tea plants using a whole-genome resequencing strategy. Previously, the domestication origin and breeding history of tea plants were mainly studied with a small number of simple sequence repeat (SSR) or single nucleotide polymorphism (SNP) markers. For instance, the comparison of the genetic diversity of Chinese and Indian tea plants with 23 pairs of SSR primers indicated the possibility of three independent domestication events for Chinese tea, Chinese CSA, and Indian CSA (Meegahakumbura et al., 2016, 2017). A total of 15,444 SNPs from 18 cultivated and wild tea accessions were identified based on RAD sequencing. Thirteen genes containing missense mutations had strong artificial selection signals (Yang et al., 2016). Genetic diversity, linkage disequilibrium, and population structure analyses were performed on 415 accessions from Guizhou Province, China, using 79,016 high-quality SNPs by the genotyping by sequencing method (Niu et al., 2019). However, due to the insufficient sample size or uneven distribution of markers, many important sites of variation or genetic regions between CSS and CSA have not been detected. To further clarify genetic differentiation and adaptive evolution of CSS and CSA, whole-genome resequencing of different tea tree cultivars is needed.

Recently, Pacbio sequencing and high-throughput/resolution chromatographic capture (Hi-C) technology has more accurately assembled the tea tree genome into 15 pseudo chromosomes with a genome size of approximately 2.94 G (Xia et al., 2020a). In the present study, the SNPs and insertion–deletion mutations (indels) of variant sites were obtained by aligning the sequenced fragments of 33 samples to the latest reference genome. These variant sites, especially those located in genetic regions, will provide important genetic resources for the functional research and breeding projects of tea trees. Novel SNP and indel patterns of tea cultivated species were revealed by comprehensive resequencing. The genetic structure of CSS and CSA was analyzed using a high confidence SNP variation site set. A large number of mutation sites unique to CSS or CSA were identified. In addition, through the evaluation of F-Statistics (Fst), some divergence genetic regions were identified from the genome. Genes located in these regions may produce functional changes in CSS and CSA, including the acquisition, silencing, enhancement, and inhibition of gene functions. Others, in this project, a large number of stable and reliable indel markers located in these genes have been developed, which will provide important support for exploring the domestication and improvement, mining functional genes, and QTL mapping of tea trees.

## Materials and Methods

### Sample Collection and Genome Resequencing

Nine cultivars of assamica tea (CSA), including “Qingshui 3,” “Yunkang 43,” “Mengkudaye,” “Menghaidaye,” “Changyebaihao,” “Yunkang 14,” “Yunkang 37,” “Shuangjiangheidaye,” and “Yunxun 9” were collected from National Tea Plant Resource Center of Yunnan Province. Fifteen cultivars of sinensis tea (CSS) and four cultivar assamica tea (“Yunkang 10,” “Zijuan,” “Yinghong 9,” and “Xiuhong”) were collected from Anhui Agricultural University Tea Plant Resource Center located in Lujiang County, Hefei City, Anhui Province. The remaining two assamica (“Nannuoshan 14” and “Hekai 33”) and three wild taliensis (CTS1, CTS2, and CTS3) tea were collected from ancient tea area in Menghai County, Yunnan Province. The above 33 germplasms were submitted for re-sequencing, and their detailed information was shown in Supplementary Table 4 and Supplementary Figure 1. The plucked leaf samples were stored at −80°C until their genomic DNA was extracted by cetyl trimethylammonium bromide and a sequencing library was constructed. Qualified genomic DNA was cut into 350 bp fragments by ultrasound to build the DNA libraries. Paired-end sequencing libraries with an insert size of 150 bp were constructed according to the manufacturer’s instructions for sequencing on the Hiseq 2,500 platform (Illumina, San Diego, CA, United States). After removing the reads with an adapter, the reads with *N* content >10% and low-quality reads (sequences whose bases with a quality value of ≤10 account for more than 50% of the total sequence length), clean reads were obtained.

### Sequence Alignment, Variant Calling From Resequencing Data, and Filtering

The clean reads were mapped to a chromosome level tea tree reference genome of ‘Shuchazao’ using BWA-MEM (version 0.7.10) with parameters “−T 20 −k 30”. Picard-1.124 was used to filter redundant reads (MarkDuplicates) to ensure the accuracy of the variant calling. The SNP and small indel variant locus were called by the GATK-4.1.8.1-HaplotypeCaller method to generate a gvcf format file for each sample. They were merged by GATK-CombineGVCFs to obtain the population variations vcf file. The mutation sites were filtered using the following criteria: (1) SNPs within 5 bp near indel and adjacent indels within 10 bp were filtered out based on a perl script included in bcftools; (2) the number of variations in the 5 bp window should not exceed 2; (3) QUAL < 30| | QD < 2.0| | MQ < 40| | FS > 60.0 was used in variant filtration in GATK-4.1.8.1, the Ts/Tv (transition/transversion), and the heterozygous rate of each sample was recorded; (4) the plink-1.9 was used to filter the population variant locus with MAF < 0.05 and missing data rate >10%.

### Population Stratification, Phylogenetic Analysis, and PCA

Considering the reliability of the analysis results, we first removed the linkage disequilibrium SNP markers using a sliding window method with plink-1.9. The window length, step size, and *r*^2^ threshold were set to 50, 10, and 0.2 bp, respectively. The newly obtained vcf files were converted into phylip, map, ped, and bed files using tassels-5.2.50 and plink-1.9. Finally, variant sites of SNPs were used for population stratification, phylogeny analysis, and PCA. The optimal subgroup *k* value tested from 2 to 8 and population stratification analysis were performed by admixture-1.3.0 using default parameters. The result was drawn using the pophelper-2.3.0 package. MEGAX software was used to build a phylogenetic tree of 33 tea germplasms with 1000 bootstrap replicates by the neighbor-joining method. The phylogenetic tree was adjusted using the FigTree-1.4.3 software. PCA was performed by GCTA-1.9.

### Genomic Diversity, Unique Mutation Sites, and Genetic Differentiation Region Whole-Genome Screening

Based on the VCFtools and SnpEff packages, the nucleotide diversity (π), Tajima’s D, and pairwise F-Statistics (Fst) values were calculated with a 500 kb window size and 50 kb step. The genomic regions with a Fst value ≥0.368 (top 5% of the Fst value) were used as differentiation bins. Unique SNPs and indels between CSA and CSS were screened and annotated separately. According to the annotated vcf file, genes with dn/ds (non-synonymous/synonymous) >1 and located in differentiation regions were picked from the genome for KEGG annotation and enrichment analysis using a web program^1^. Selective scan analysis was performed as previously described (Xia et al., 2020a).

### Primer Design and Validation of the Indel Markers

To verify the accuracy of genotyping and develop indel markers for genetic research, indels with a length ≥5 bp were used as candidate loci. A total of 120 unique primer pairs were designed based on the flanking sequences of indel loci through the Primer 3.0 program. The length of the primers was set as 20 to 22 with an optimum length of 21 bp. The primer pairs were preliminary screened on eight tea cultivars, including four CSS (“Anhui 3,” “Longjing 43,” “Tieguanyin,” and “Shancha 1”) and four CSA (“Hekai 33,” “Nanuoshan 14,” “Yunkang 10,” and “Zijuan”). The primer pairs with polymorphism and unambiguous amplification bands were screened by polymerase chain reaction (PCR) and the Fragment Analyzer 96 (Advanced Analytical Technologies, Inc., Ames, IA, United States) for further identification of 30 tea samples. Detailed information of the 30 tea samples is provided in Supplementary Table 5. Subsequently, according to the PCR amplification results, *He* and *Ho* were calculated using Popgene32 software. The number of alleles (*Na*), *MAF*, and *PIC* were calculated using PowerMarker3 software. A more detailed approach was described in previous studies (Liu et al., 2018, 2019). Transcriptome data used in this study were downloaded from NCBI (PRJNA52233).

## Results

### Detection of Genome-Wide Variant Sites

Genomes of 33 tea accessions, including 15 CSS and 15 CSA, from 14 provinces in China were sequenced using the Hiseq2500 platform. Three wild taliensis tea (CTS1, CTS2, and CTS3) were used as the outgroup (Table 1). A total of 1570 Gb high-quality clean reads were obtained with an average Q30 value of 91.3% and average GC content of 39.4%. Approximately 48 Gb of clean data was obtained for each sample, with an average sequencing depth of 16 and 1× coverage of more than 88.7% (Supplementary Table 1). These high-quality sequences were aligned to the chromosome level high-precision genome (Xia et al., 2020a). The average mapping rate produced 96.53% alignment and proper mapping reached 84.68% (Supplementary Table 1).

**TABLE 1:** Summary of variants from all tea samples.

Name	SNP	INDEL	SUM	Ho	He	Ts/Tv	Non-syn/Syn
Anhui 3	3676324	1633262	5309586	0.13	0.22	2.68	1.65
Fudingdabai	4423650	1872696	6296346	0.19	0.22	2.67	1.65
Longjing 43	3700123	1655737	5355860	0.13	0.22	2.67	1.64
Baihaozao	3861305	1721101	5582406	0.14	0.22	2.67	1.65
Xinyang 10	3641455	1626369	5267824	0.13	0.22	2.67	1.64
Guihong 3	3745749	1673269	5419018	0.13	0.22	2.67	1.66
Echa 5	4012657	1772592	5785249	0.15	0.22	2.67	1.66
Chuanmu 28	3889553	1727255	5616808	0.15	0.22	2.66	1.66
Xicha 5	3781285	1703838	5485123	0.13	0.22	2.67	1.66
Gancha 3	4012013	1754996	5767009	0.15	0.22	2.69	1.65
Qucha	3568154	1607688	5175842	0.12	0.22	2.66	1.66
Shancha 1	3844480	1708124	5552604	0.13	0.22	2.69	1.64
Xiaoxianghong 21-3	4151438	1760118	5911556	0.15	0.22	2.71	1.62
Zhenong 117	3971472	1729607	5701079	0.14	0.22	2.67	1.64
Yaoshanxiulv	3737713	1677786	5415499	0.13	0.22	2.66	1.65
Yunkang 10	4536915	1869963	6406878	0.17	0.22	2.67	1.62
Zijuan	4567906	1903947	6471853	0.17	0.22	2.67	1.62
Xiuhong	4131555	1601387	5732942	0.16	0.22	2.72	1.60
Yinghong 9	4448683	1731791	6180474	0.17	0.21	2.69	1.61
Qingshui 3	4272540	1667201	5939741	0.16	0.22	2.72	1.61
Changyebaihao	4129310	1653708	5783018	0.12	0.21	2.70	1.60
Yunkang 43	4223486	1717369	5940855	0.14	0.21	2.69	1.61
Yunxuan 9	4143841	1694662	5838503	0.11	0.21	2.68	1.62
Shuangjiangheidaye	4170410	1699774	5870184	0.11	0.21	2.69	1.61
Menghaidaye	4180475	1657480	5837955	0.12	0.21	2.70	1.62
Mengkudaye	4887656	1852883	6740539	0.21	0.22	2.71	1.62
Yunkang 14	4229040	1683084	5912124	0.14	0.21	2.70	1.62
Yunkang 37	4296614	1739395	6036009	0.14	0.21	2.69	1.61
Nannuoshan 14	4300939	1760193	6061132	0.12	0.21	2.68	1.62
Hekai 33	4108626	1659334	5767960	0.11	0.21	2.68	1.62
CTS-1	3584397	1377392	4961789	0.08	0.20	2.588	1.00
CTS-2	3579822	1347100	4926922	0.08	0.20	2.61	1.00
CTS-3	3589847	1374588	4964435	0.08	0.20	2.592	1.00

Among the 33 tea accessions, 18,903,625 SNPs were identified with an average density of 8.5/kb. Except for the 1,116,245 multiple allele SNPs (5.9%), the remaining 17,787,380 were classified into transitions (Ts: G/A and C/T) and transversions (Tv: A/C, A/T, C/G, and G/T). Transitions and transversions accounted for 67.6% (12,785,494) and 26.5% (5,001,886) of the total number of SNPs, respectively, with a transition/transversion (Ts/Tv) ratio of 2.56. A total of 7,314,133 indels were identified, including 2,095,200 insertion variants, 3,784,855 deletion variants, and 1,434,078 multiple allele indel loci, with an average indel density of 4.9/kb. The number of mutation sites contained in different samples varied from the lowest of 4,926,922 to the highest of 67,405,39 (Table 1). Calculations based on SNP mutations showed that, except for observed heterozygosity *(Ho*) with large variations with values of 0.08 to 0.21, the expected heterozygosity (*He*), Ts/Tv, and non-synonymous/synonymous (Non-syn/Syn) ratios were almost equal for all cultivated varieties (Table 1).

**FIGURE 1:**
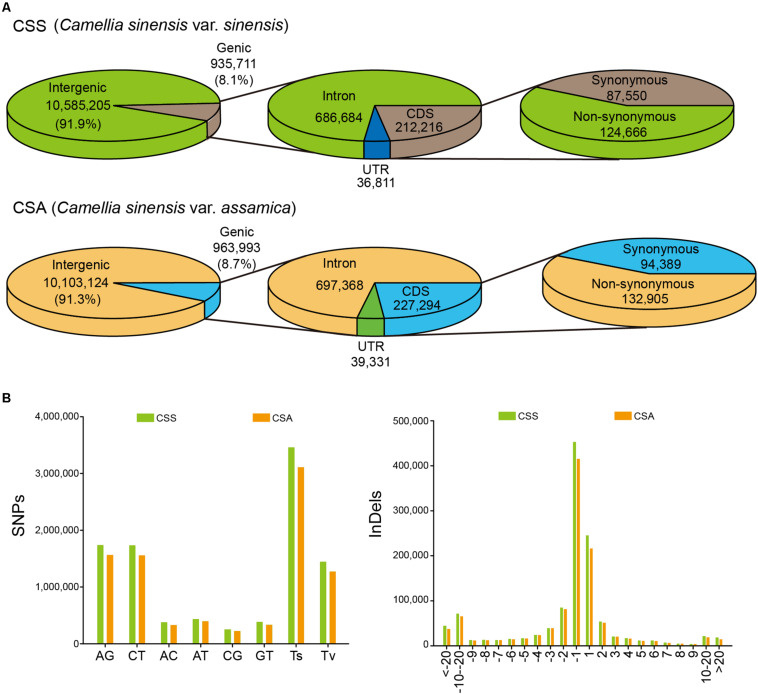
Classification and distribution of identified SNPs/indels among 30 samples. **(A)** Annotation of SNPs identified in CSS and CSA subgroup. **(B)** The mutation pattern (SNP) and the length distribution (indel) of unique variations for CSS and CSA subgroup, respectively.

According to the genetic background, in addition to the three CTS as the outgroup, all other samples were divided into two subgroups of CSS and CSA, with 15 samples in each subgroup. The number of SNPs in the intergenic region and single nucleotide indels were predominantly large in each subgroup. Although the average number of SNPs (3,867,825) and indels (1,708,296) in the CSS subgroup for each individual was smaller than the average (4,308,533 SNPs and 1,726,145 indels) of each individual in the CSA subgroup, the total SNPs (13,034,839) and indels (4,326,311) were more abundant than the CSA subgroup (12,507,724 SNPs and 4,189,516 indels). These findings suggested that the CSS subgroup had a larger number of rare alleles. However, for the CSA subgroup, there were more SNPs located in the genic region (963,993) and non-synonymous mutations (132,905 non-synonymous mutations with a Non-syn/Syn ratio of 1.408), while the Non-syn/Syn ratio (1.424) of the CSS subgroup was only slightly and insignificantly higher (Figure 1). A large number of unique variations were found between CSS (5,099,732 unique SNPs and 1,340,687 unique indels) and CSA (4,572,617 unique SNPs and 1,603,893 unique indels). These loci showed similar patterns in the two subgroups (Figure 1).

### Phylogenetic and Population Structure Analyses of Tea Germplasms

**FIGURE 2:**
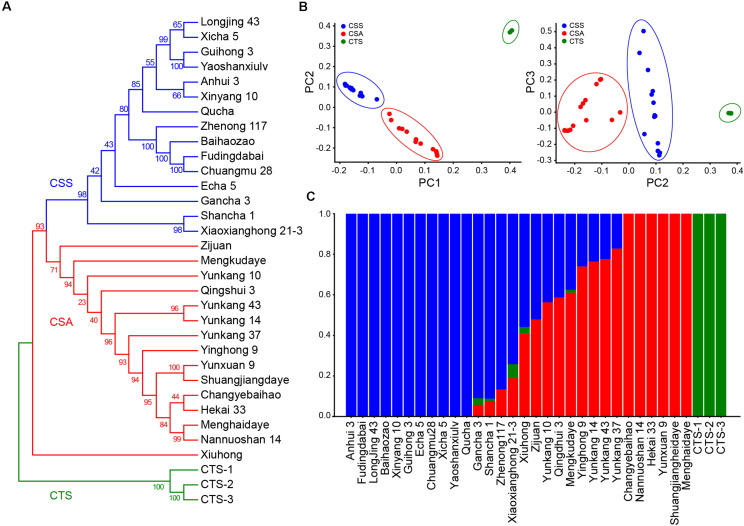
Phylogenetic, population stratification analysis, and Principal Component Analyses (PCA) based on 5,52,750 SNPs. **(A)** Neighbor-Joining phylogenetic tree of 33 tea germplasms. **(B)** PCA analysis for 33 tea samples. **(C)** Population stratification based on admixture analysis when K set as three.

To evaluate the population stratification and genetic relationship of tea germplasms at the genome-wide scale, phylogenetic tree analysis, admixture analysis, and Principle Component Analysis (PCA) were performed based on 5,52,750 non-linkage disequilibrium SNPs (Figure 2). The result of the cross-check provided by admixture showed that the cross-check error had a minimum value when *k* = 3. According to this, three pure-blooded CTSs were allocated to the first group. The remaining 15 CSS and 15 CSA germplasms were classified into the second and third groups. In the CSS and CSA subgroups, 13 exhibited an admixed ancestry, including 9 assamica (“Xiuhong,” “Zijuan,” “Yunkang 10,” “Qingshui 3,” “Mengkudaye,” “Yinghong 9,” “Yunkang 14,” “Yunkang 43,” and “Yunkang 37”) and 4 sinensis (“Gancha 3,” “Shancha1,” “Xiaoxianghong21-3,” and “Zhenong 117”). The remaining 6 assamica (“Changyebaihao,” “Nannuoshan 14,” “Hekai 33,” “Yunxuan 9,” “Menghaidayezhong,” and “Shuangjiangdaheiye”) and 11 sinensis (“Anhui 3,” “Fudingdabai,” “LongJing 43,” “Baihaozao,” “Xinyang 10,” “Guihong 3,” “Echa 5,” “Chuanmu 28,” “Xicha 5,” “Yaoshanxiulv,” and “Qucha”) exhibited pure ancestry (Figure 2C). The PCA results of different dimensions demonstrated that the 33 samples could also be divided into three clusters, which was very consistent with the admixture analysis, reflecting the existence of a strong genetic differentiation (Figure 2B).

The phylogenetic analysis showed that the clustering patterns obtained by the three analyses were highly consistent. CSS species grown in tropical and subtropical regions were classified into the first category, and CSA species grown in tropical regions were classified into the second category. These classifications were highly consistent with their geographical distributions (Figure 2). The phylogenetic analysis revealed a complex interspecific relationship of CSS or CSA. “Xicha 5” from Jiangsu Province and “Longjing 43” from Zhejiang Province were clustered into one group with a bootstrap support of 65, “Anhui 3” from Anhui Province and Henan Province “Xinyang 10” was clustered into one group with a bootstrap support of 66, “Zhennong 117” with the blood of “Fudingdabai” from Zhejiang Province, “Baihaozao” from Hunan Province, and “Fudingdabai” from Fujian Province and “Chuanmu 28” from Sichuan Province were closely clustered into one group with a bootstrap support of 100. These findings implied that tea varieties from different regions may share the same parent. Interestingly, two tea varieties (“Guihong 3” and “Yaoshanxiulv”) from Guangxi Province closely clustered into one group. One of two tea varieties from Guangdong Province formed a single clade (“Xiuhong”) and the other variety (“Yinghong 9”) as well tea varieties from Yunnan Province were tightly clustered with a bootstrap support of 94 (Figure 2A). These findings suggested a different genetic origin, even for varieties from the same cultivated area.

### Genomic Genetic Differentiation and Evolutionary Patterns Between CSS and CSA

**FIGURE 3:**
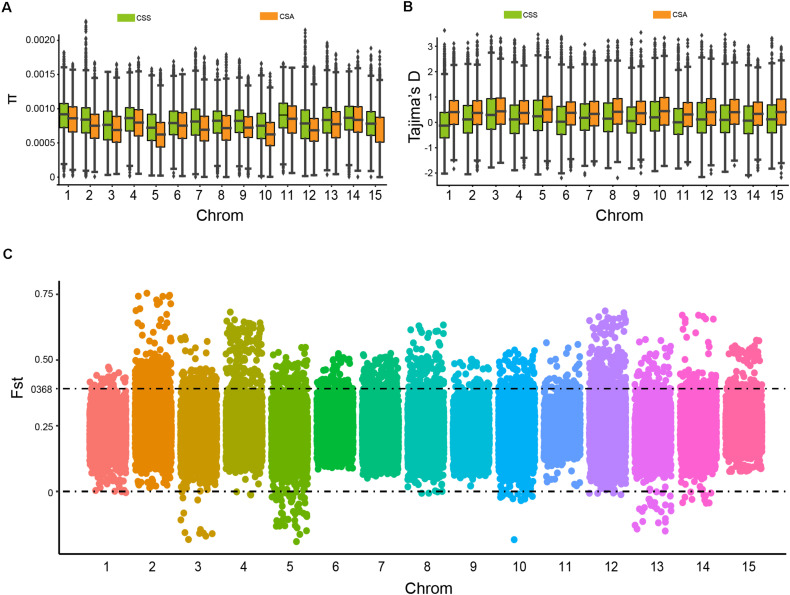
The nucleic acid diversity (π), Tajima’s D value of CSS and CSA subgroups, and candidate differentiation regions identified in CSS and CSA subgroups on the genome. **(A)** Nucleic acid diversity. **(B)** Tajima’s D value. **(C)** Distribution of windowed Fst values for CSA versus CSS (window size 500 kb and step 50 kb), the dots above the dotted line represent candidate differentiation windows on the chromosome.

To further evaluate the genetic diversity and population differentiation of CSS and CSA, we calculated the nucleotide diversity (π) and Tajima’s *D* values (Figure 3). The CSS group showed slightly higher nucleotide diversity (π mean = 0.744 × 10^3^, median = 0.776 × 10^3^, and std = 0.329 × 10^3^) than the CSA group (π mean = 0.683 × 10^3^, median = 0.683 × 10^3^, and std = 0.316 × 10^3^). This result was consistent with the number of mutation sites they contained. Interestingly, the values of π and Tajima’s D showed opposite trends, compared with CSS (Tajima’s D mean = 0.208, median = 0.143, and std = 0.838), and higher Tajima’s D in CSA (mean = 0.436, median = 0.421, and std = 0.749), implying stronger balancing selection and lower rare allele frequencies.

**FIGURE 4:**
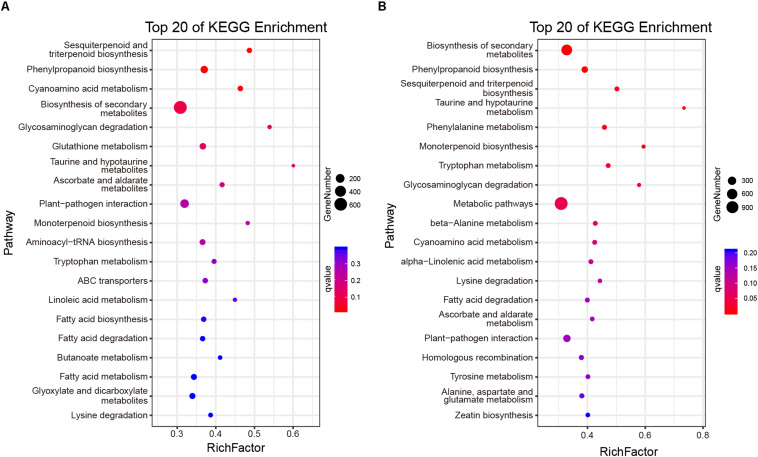
KEGG functional categorization of the positive selection gene. **(A)** Enrichment analysis of positive selection genes of CSS subgroups. **(B)** Enrichment analysis of positive selection genes of CSA subgroups.

At the same time, the ratio of non-synonymous and synonymous mutations (dN/dS = ω) was calculated to explore the evolutionary direction of genes (Xanthopoulou et al., 2019; Ogutu et al., 2020). When ω > 1, 14,594 and 14,865 genes were selected from CSS and CSA subgroups, respectively. These genes were considered to have undergone positive selection. When ω < 1, 7,202, and 7,414 genes from CSS and CSA, respectively, that may have undergone purification selection were found. Kyoto Encyclopedia of Genes and Genomes (KEGG) of the positively selected genes revealed that genes related to tea quality, such as terpenes, phenylalanine, and other secondary metabolites biosynthesis, were significantly enriched in CSS or CSA subpopulations (Figure 4).

In addition, to estimate population differentiation and to screen differentiated genetic regions between the two groups, pairwise F-Statistics (Fst) values (Fst mean = 0.2336, median = 0.2285, and std = 0.0825) were calculated based on the whole genomic SNP data. The degree of differentiation of the two subpopulations differed in 15 putative chromosomes. It should be noted that Fst may be <0 when the degree of variation in the subpopulation is greater than that of the whole population (Figure 3C). When the threshold was set to 0.368 (top 5% of the Fst value), 5121 genes (10.3% of the total genes in tea plant) contained in 2959 bins were identified as candidate genes. Subsequently, KEGG enrichment analysis of these genes revealed that the top 20 KEGG enrichment pathways involved plant hormone signal transduction, pyruvate metabolism, cutin, suberin and wax biosynthesis, anthocyanin biosynthesis, photosynthesis, and others (Supplementary Figure 2).

### Selection Sweep Between CSS and CSA

**FIGURE 5:**
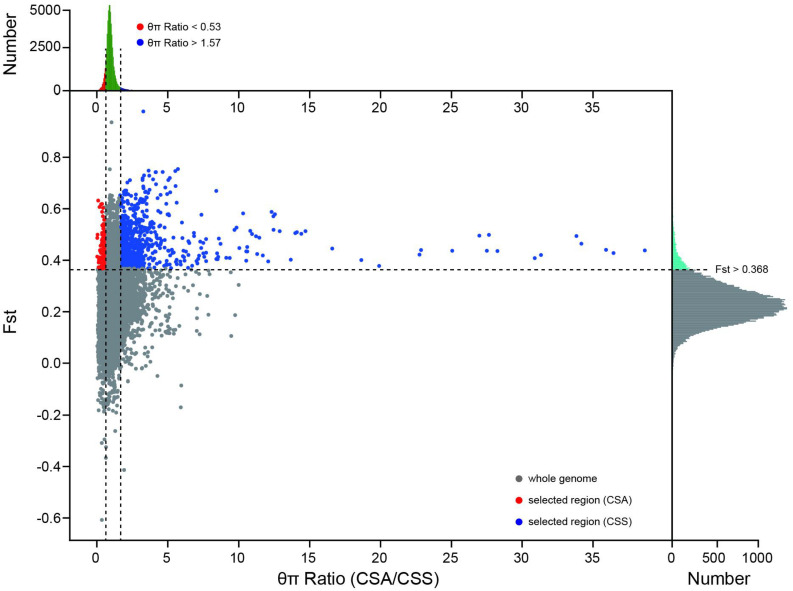
Fst and θπ based selective sweep identification between CSS and CSA population. The red dots and blue dots indicate the selected regions of the CSA and CSS subgroups on the genome, respectively. The subplot on the top reflects the distribution of θπ Ratio, and the subplot on the right reflects the distribution of Fst.

Adaptation to nature and artificial selection will leave traces on the gene pool of the population. Xia et al. identified 510 windows and 1569 genes related to domestication and improvement (Xia et al., 2020a). The selection region between CSS and CSA subsets has not yet been identified. Based on Fst and θπ for selective sweeping analysis, we identified 830 and 141 selection regions in CSS and CSA populations, which contained 687 and 124 genes, respectively (Figure 5). Only 85 genes were described in previous studies. Further functional annotation of these genes revealed their involvement in environmental adaptation, metabolism, and genetic information processing. Some of these genes that are related to growth and development, such as carotenoid synthesis, cell division, photorespiration, and stress resistance, may lead to different environmental adaptability and morphological characteristics of the two subgroups. Detailed selection regions and gene information are presented in Supplementary Table 2.

### Development of Indel Markers Related to Genetic Differentiation

**FIGURE 6:**
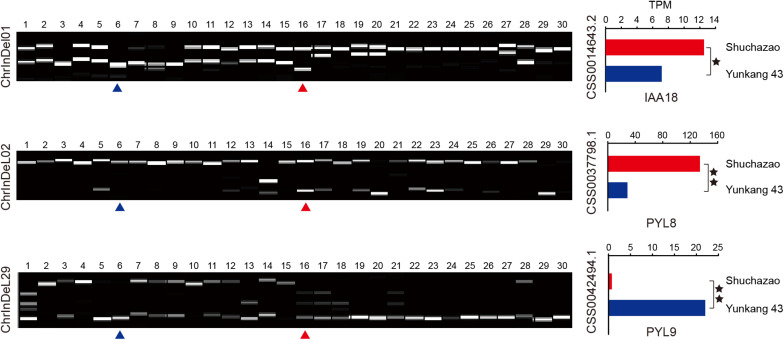
Exhibition of transferability and polymorphism of three hormone-related indel markers among 30 tea varieties. Code 1∼15 and code 28 represent CSA varieties, others represent CSS varieties. Blue arrow and red arrow represent “Yunkang 43” and “Shuchazao” varieties, respectively. TPM (Transcripts Per Kilobase of exon model per Million mapped reads) represents the expression level of the gene they are located in.

We randomly selected 120 indel loci located in the differentiation region to develop indel markers. These primers were preliminarily screened with eight tea varieties. After removing 34 non-polymorphic and 33 ineffective amplification, 53 primer pairs with clear polymorphic amplification bands were successfully selected and further applied to 30 tea varieties to detect their cross-varieties/species transfer capability (Figure 6 and Supplementary Figure 3).

Among them, 24, 20, and 9 indel markers showed high polymorphism, moderate polymorphism, and low polymorphism in 30 tea varieties, respectively (Zhang et al., 2017). The polymorphism information content (*PIC*) values ranged from the lowest 0.0624 (ChrInDel16) to the highest 0.7883 (ChrInDeL11), with an average of 0.4494. In addition, the major allele frequency (MAF), number of alleles (Na), observed heterozygosity (Ho), and expected heterozygosity (*He*) were also used to evaluate newly developed indel markers (Table 2). The MAF values ranged from 0.30 (ChrInDeL11, ChrInDel35) to 0.9667 (ChrInDeL16) with an average value of 0.6050. The Na values ranged from 2 (ChrIndeL15, ChrIndeL16, ChrIndeL21, ChrIndeL24, ChrIndeL30, ChrIndeL32, ChrIndeL33, and ChrInDeL53) to 8 (ChrInDeL12 and ChrIndeL13) with an average of 4. *Ho* ranged from 0 (ChrIndeL16 and ChrIndeL35) to 1 (ChrInDeL42 and ChrInDel51) with an average value of 0.449. *He* ranged from 0.0655 (ChrInDeL16) to 0.8266 (ChrInDeL11) with an average value of 0.5137. It should be pointed out that the *PIC* and *He* values of these markers were not only similar but also have the same change trend, while the change trend of *MAF* was exactly opposite to this trend. In addition, some markers had higher application value due to different allele patterns in the CSS and CSA subgroups. For example, ChrInDeL01 correctly divided the 30 samples used for marker verification into the CSS and CSA subgroups. ChrInDeL02 and ChrInDeL29 had opposite allelic distribution characteristics and the expression levels of the genes in which they are located differed significantly between the two CSS and CSA cultivars (Figure 6). More detailed genetic information and amplification of these newly developed markers are shown in Table 2 and Supplementary Figure 3. The sequences of 53 primer pairs and their genomic positions are listed in Supplementary Table 3. Using these newly developed indel markers, we performed phylogenetic construction on 30 tea plants. These 30 tea plant varieties were clearly divided into the CSS and CSA subgroups in a manner that was strongly consistent with their breeding history, geographic distribution, and leaf shape characteristics (Supplementary Figure 4). These results suggested that the markers have high polymorphism and good mobility, and can be valuable for molecular-assisted breeding, fingerprinting, and functional research.

**TABLE 2:** Characteristics of 53 newly developed indel markers.

Marker ID	Region	MAF	Na	PIC	Ho	He
ChrInDeL01	exon_variant	0.4500	6	0.6452	0.6	0.7045
ChrInDeL02	3_prime_UTR_variant	0.7333	3	0.3468	0.3333	0.4136
ChrInDeL03	3_prime_UTR_variant	0.6333	4	0.4599	0.1333	0.5311
ChrInDeL04	5_prime_UTR_variant	0.4667	3	0.5564	0.6667	0.6418
ChrInDeL05	disruptive_inframe_deletion	0.7833	5	0.3535	0.2333	0.378
ChrInDeL06	disruptive_inframe_insertion	0.4167	6	0.7144	0.7333	0.7593
ChrInDeL07	downstream_gene_variant	0.5500	3	0.3956	0.8667	0.5181
ChrInDeL08	downstream_gene_variant	0.7000	3	0.3929	0.1333	0.4588
ChrInDeL09	downstream_gene_variant	0.4833	4	0.5016	0.1667	0.5972
ChrInDeL10	downstream_gene_variant	0.4000	6	0.6777	0.8333	0.735
ChrInDeL11	frameshift_variant&start_lost	0.3000	7	0.7883	0.1667	0.8266
ChrInDeL12	frameshift_variant&start_lost	0.3500	8	0.7233	0.7	0.7712
ChrInDeL13	frameshift_variant&start_lost	0.5000	8	0.6475	0.5	0.6949
ChrInDeL14	frameshift_variant&start_lost	0.6333	5	0.5117	0.3	0.5605
ChrInDeL15	frameshift_variant&stop_gained	0.5167	2	0.3747	0.9	0.5079
ChrInDeL16	frameshift_variant&stop_gained	0.9667	2	0.0624	0	0.0655
ChrInDeL17	intergenic_region	0.6667	4	0.4011	0.2667	0.4819
ChrInDeL18	intergenic_region	0.5833	4	0.4311	0.8333	0.5328
ChrInDeL19	intergenic_region	0.5333	3	0.5258	0.6	0.6079
ChrInDeL20	intergenic_region	0.3667	7	0.7397	0.6333	0.7842
ChrInDeL21	intergenic_region	0.8333	2	0.2392	0.2	0.2825
ChrInDeL22	intergenic_region	0.5833	4	0.5043	0.7	0.578
ChrInDeL23	intergenic_region	0.5833	4	0.5205	0.5	0.5881
ChrInDeL24	intergenic_region	0.8667	2	0.2044	0.2667	0.235
ChrInDeL25	intergenic_region	0.9167	3	0.1500	0.1	0.1588
ChrInDeL26	intergenic_region	0.5000	5	0.5297	0.9667	0.6153
ChrInDeL27	intergenic_region	0.5000	6	0.6101	0.7333	0.6706
ChrInDeL28	intron_variant	0.6667	4	0.4416	0.2667	0.504
ChrInDeL29	intron_variant	0.6167	3	0.4686	0.5	0.5441
ChrInDeL30	intron_variant	0.9500	2	0.0905	0.1	0.0966
ChrInDeL31	intron_variant	0.5000	7	0.5718	0.3333	0.6429
ChrInDeL32	intron_variant	0.9167	2	0.1411	0.0333	0.1554
ChrInDeL33	intron_variant	0.8833	2	0.1849	0.2333	0.2096
ChrInDeL34	intron_variant	0.5333	4	0.4199	0.9	0.5362
ChrInDeL35	intron_variant	0.3000	5	0.7108	0	0.7661
ChrInDeL36	intron_variant	0.4333	4	0.6019	0.1333	0.678
ChrInDeL37	intron_variant	0.6667	4	0.4833	0.3333	0.5266
ChrInDeL38	intron_variant	0.4667	4	0.4644	0.9667	0.5712
ChrInDeL39	start_lost&conservative_inframe_deletion	0.4500	6	0.5272	0.9333	0.6164
ChrInDeL40	start_lost&conservative_inframe_deletion	0.9333	3	0.1213	0.0667	0.1282
ChrInDeL41	start_lost&conservative_inframe_deletion	0.9500	3	0.0936	0.0333	0.0977
ChrInDeL42	stop_gained&conservative_inframe_insertion	0.4833	5	0.5026	1	0.5977
ChrInDeL43	stop_gained&conservative_inframe_insertion	0.6500	3	0.3902	0.6333	0.4842
ChrInDeL44	upstream_gene_variant	0.4000	3	0.5837	0.6667	0.6689
ChrInDeL45	upstream_gene_variant	0.6833	3	0.4030	0.0333	0.474
ChrInDeL46	upstream_gene_variant	0.5667	5	0.5572	0.1	0.6164
ChrInDeL47	upstream_gene_variant	0.5500	3	0.5246	0.1	0.6028
ChrInDeL48	upstream_gene_variant	0.4667	3	0.5564	0.0667	0.6418
ChrInDeL49	upstream_gene_variant	0.7667	4	0.3697	0.4333	0.3994
ChrInDeL50	upstream_gene_variant	0.6500	3	0.4425	0.4	0.5136
ChrInDeL51	upstream_gene_variant	0.5000	3	0.3990	1	0.5249
ChrInDeL52	upstream_gene_variant	0.7500	4	0.3847	0.5	0.4198
ChrInDeL53	upstream_gene_variant	0.5167	2	0.3747	0.9667	0.5079

## Discussion

A long breeding history usually reduces the genetic diversity of crops. Therefore, it is important to study the origin and domestication of species for crop improvement and domestication (Zsogon et al., 2018). Since no credible wild ancestor of CSS has been found, it is generally believed that CSS originated from southwest China and underwent independent domestication to adapt to various living environments (Meegahakumbura et al., 2016). However, it is puzzling that cultivated tea plants showed higher genetic diversity than wild tea plants in previous studies (Yang et al., 2016; Niu et al., 2019). More critically, important differentiation regions and genes of CSS and CSA variants in their genomes have not been identified. In this study, 15 CSS, 15 CSA, and 3 CTS tea varieties were subjected to high-depth whole-genome resequencing to explore the evolutionary patterns of the different subpopulations. After filtering out low-quality reads, a total of 1570 Gb of high-quality resequencing clean data was obtained, with an average depth of 16× for each sample.

By aligning clean reads to the reference genome, and then detecting and strictly filtering mutation sites with various software, we finally identified 18,903,625 SNPs and 7,314,133 indels among 33 tea varieties, with average SNP and indel densities of 8.5 and 4.9/kb, respectively. In the process of evolution, structurally similar bases are easily replaced, and short indel mutations are less harmful to species (Yang and Yoder, 1999; Saxena et al., 2014). Therefore, the most abundant types of SNP and indel mutations are transition (Ts: G/A and C/T) and single nucleotide indels, respectively. Similar research results have been reported in rice (Li et al., 2018), cotton (Shen et al., 2017), tomato (Causse et al., 2013), quinoa (Zhang et al., 2017), mume and other crops (Zhang et al., 2018). It should be noted that for each sequenced sample, *He*, Ts/Tv, and the ratio of non-synonymous substitutions to synonymous substitutions were almost the same, except that *Ho* was different (from 0.08 to 0.21). This may be caused by long-term interspecific hybridization and suggests that different tea tree varieties may have common ancestry and origin. Compared with other plants, such as pear (Wu et al., 2018) and soybean (Lam et al., 2010), we observed that the ratio of non-synonymous substitutions to synonymous substitutions of tea trees was higher. This ratio is comparable to millet (Bai et al., 2013) and, but lower than that of lotus (Liu et al., 2016).

Mutation sites in genic regions may have important effects on plant growth and development (Jin et al., 2016, 2018; Xanthopoulou et al., 2019; Wang et al., 2020). For example, a single nucleotide mutation in gibberellic acid receptor GID1 could cause a gibberellic acid-insensitive dwarf phenotype in peaches (Cheng et al., 2019). Shang et al. (2014) reported that SNP-1601, located upstream of the start codon of the gene Csa5G157230 can regulate the bitter taste of cucumber through extensive resequencing and genome-wide association study. Presently, up to 91.5% of SNPs were determined to be located in intergenic regions, with only 8.5% of SNPs located in genes, and 329,698 SNPs located in CDS regions were annotated as non-synonymous mutations. Thus, tea plants may have diversified phenotypes and adaptability. Up to 6,440,419 and 5,776,510 unique mutations were identified in CSS and CSA subpopulations, respectively, indicating that the two subpopulations have undergone extensive genetic differentiation at the genome-wide level. Interestingly, compared with the CSA subgroup, the total number of mutation loci in the CSS subgroup was larger, while each sample contained fewer mutation sites on average. One possible explanation is that the samples in the CSS subgroup have a wider geographical origin, and the complex natural environment drives the formation of more adaptive mutations. The data will be important in studies to genetically improve tea trees.

Cultivars of sinensis tea and CSA are the two most important tea tree varieties. They vary widely in geographical distribution, morphological characteristics, and flavor substance content. Generally, CSS, which features small leaf morphology and higher cold resistance, is widely distributed in tropical and subtropical regions. CSA, with larger leaves and stronger growth but lower cold resistance, is distributed in tropical regions. Long-term outcrossing has formed a complex blood relationship between CSS and CSA (Zhao et al., 2014a; Xia et al., 2017; Wei et al., 2018; Liu et al., 2019). In this study, we used the high confidence of 552,750 non-linkage disequilibrium SNPs obtained from sequencing to perform population structure analysis, PCA, and phylogenetic tree construction of 33 samples to evaluate their genetic diversity. Despite different evaluation strategies being used, the 33 samples were clearly divided into three subgroups of CSS, CSA, and CTS, which proved the accuracy of our genotyping. In the CSS and CSA subgroups, 4 and 9 varieties were mixed in the pedigree analysis (Figure 2). This indicated frequent gene communication between the CSS and CSA subgroups. Their genetic background was obviously different from that of the CTS subgroup.

The domestication history of tea plants has not been clarified. It is generally believed that to improve the flavor of tea, people have only made changes to very limited number regions and these regions occupy a very low ratio in the genome. The significant difference between CSS and CSA may be mainly affected by natural selection. By calculating the π, Tajima’s D, and Fst values of CSS and CSA subgroups independently, we found that the nucleic acid diversity of the CSA (π mean = 0.683 × 10^3^) subgroup was slightly lower than that of CSS (π mean = 0.744 × 10^3^), but was significantly higher than that of pear and cotton (Du et al., 2018; Wu et al., 2018). Interestingly, the CSA subgroup displayed a higher Tajima’s D value (mean = 0.436) and the Tajima’s *D*-value of the CSS subgroup (mean = 0.208) was approximately half that of CSA, indicating that the CSA subgroup lacks rare alleles due to stronger natural selection. This is probably because the CSA tea varieties in China are mainly cultivated in fewer provinces that feature similar climatic conditions. The 2959 differentiation and 971 selection regions identified from the genome will inform further studies to understand the genetic differentiation and environmental adaptability between the CSS and CSA subgroups. Enrichment analysis revealed that auxin-associated genes or pathways accounted for the highest abundance, suggesting that there may be different growth and development mechanisms between CSS and CSA species because they play important roles in plant root growth, leaf development, and biological and abiotic resistance.

Hormones play an indispensable role in the processes of plant growth and resistance to stress (Brian, 1978; Dreher and Callis, 2007; Blázquez et al., 2020). Tea trees with small leaves cultivated in subtropical regions stop growing due to the cold and reduced light time, while tea trees cultivated in tropical regions maintain a strong growth potential and sprout young shoots. The plant hormone signal transduction pathway contains some of the genes involved in root (GO:0048767 and GO:0010086) and leaf (GO:0048366, GO:0010150, and GO:0048831) development, ultraviolet (UV) and far-red light response (GO:0010224 and GO:0010218), cold (GO:0009409 and GO:0070417), and drought resistance (Supplementary Figure 2). We speculate that these genes may be related to the developmental regulation and ecological adaptability of the two subpopulations. For instance, UV-B light and the abscisic acid receptor PYL8 can regulate cotyledon root development and stress adaptation by MYB13 interaction (Zhao et al., 2014b; Qian et al., 2020). The abscisic acid receptor PYL9 improves drought resistance by regulating leaf senescence (Zhao et al., 2016). Transcriptome analysis of tea trees has revealed that the plant hormone signal transduction pathway contains many key genes in response to cold treatment (Zheng et al., 2016; Ban et al., 2017; Hao et al., 2018). Interestingly, we found that PYL8 and PYL9 showed significant expression differences in some CSS and CSA varieties, and the distinct genotype characteristics of the two indel markers (ChrInDeL02 and ChrInDeL29) may cause the two genes to show opposite expression trends, thus regulating the growth potential and environmental adaptability of CSS and CSA (Figure 6). In addition, a unique indel mutation was identified in the exon of the AUX/IAA18 gene (relating to plant root development) between the CSS and CSA varieties (Figure 6 and Supplementary Figure 3), implying that IAA18, PYL8, and PYL9 may co-regulate the growth and development of the root system of tea plants (Wang et al., 2005; Ploense et al., 2009; Notaguchi et al., 2012). Although many mutation sites have not been verified, the results provide important data and clues to understand the genetic differentiation and application of marker-assisted selection of tea plants.

Molecular markers are widely used in genetic map construction, map-based cloning, and population diversity analysis. As a third-generation molecular marker, indels have the characteristics of wide genome distribution, high polymorphism, and good reproducibility (Branham et al., 2018; Deokar et al., 2019). Through preliminary screening, 53 indel markers were selected. They were able to amplify clear polymorphic bands in 30 tea varieties (Supplementary Figure 3). The average *PIC* content of these indel markers was 0.451, slightly lower than previously developed SSR markers (Liu et al., 2017). Nonetheless, they are more valuable considering that many markers are located in introns or annotated as non-synonymous mutations (Liu et al., 2018). These markers were used for population structure analysis and phylogenetic tree construction. CSS and CSA varieties were correctly divided into two categories (Supplementary Figure 4), which proves that these newly developed markers have sufficient identification for different tea varieties and can be applied to more extensive genetic research.

## Data Availability Statement

The datasets presented in this study can be found in online repositories. The names of the repository/repositories and accession number(s) can be found below: https://www.ncbi.nlm.nih.gov/, PRJNA597714.

## Author Contributions

YA, SL, and CW conceived and designed the research project. YA and XM performed data analysis and manuscript drafting. YA conducted DNA extraction, primer design, PCR amplification, SNP, and indel marker validation. XM, SZ, XX, and RG were involved in sample collection and data analysis. All authors read and approved the manuscript.

## Conflict of Interest

The authors declare that the research was conducted in the absence of any commercial or financial relationships that could be construed as a potential conflict of interest.

## Footnotes

1 https://www.omicshare.com/tools/

## References

[B1] Bai H. Cao Y. Quan J. Dong L. Li Z. Zhu Y. (2013). Identifying the genome-wide sequence variations and developing new molecular markers for genetics research by re-sequencing a Landrace cultivar of foxtail millet. *PLoS One* 8:e73514. 10.1371/journal.pone.0073514 24039970 PMC3769310

[B2] Ban Q. Wang X. Pan C. Wang Y. Kong L. Jiang H. (2017). Comparative analysis of the response and gene regulation in cold resistant and susceptible tea plants. *PLoS One* 12:e0188514. 10.1371/journal.pone.0188514 29211766 PMC5718485

[B3] Blázquez M. A. Nelson D. C. Weijers D. (2020). Evolution of plant hormone response pathways. *Annu. Rev. Plant Biol.* 71 327–353. 10.1146/annurev-arplant-050718-100309 32017604

[B4] Branham S. E. Levi A. Katawczik M. Fei Z. Wechter W. P. (2018). Construction of a genome-anchored, high-density genetic map for melon (*Cucumis melo* L.) and identification of Fusarium oxysporum f. sp. melonis race 1 resistance QTL. *Theor. Appl. Genet.* 131 829–837. 10.1007/s00122-017-3039-5 29372283

[B5] Brian P. W. (1978). Review lecture: hormones in healthy and diseased plants. *Proc. R. Soc. Lond. B Biol. Sci.* 200 231–243. 10.1098/rspb.1978.0018

[B6] Causse M. Desplat N. Pascual L. Le Paslier M.-C. Sauvage C. Bauchet G. (2013). Whole genome resequencing in tomato reveals variation associated with introgression and breeding events. *BMC Genomics* 14:791. 10.1186/1471-2164-14-791 24228636 PMC4046683

[B7] Cheng J. Zhang M. Tan B. Jiang Y. Zheng X. Ye X. (2019). A single nucleotide mutation in GID1c disrupts its interaction with DELLA1 and causes a GA-insensitive dwarf phenotype in peach. *Plant Biotechnol. J.* 17 1723–1735. 10.1111/pbi.13094 30776191 PMC6686139

[B8] Deokar A. Sagi M. Tar’an B. (2019). Genome-wide SNP discovery for development of high-density genetic map and QTL mapping of ascochyta blight resistance in chickpea (*Cicer arietinum* L.). *Theor. Appl. Genet.* 132 1861–1872. 10.1007/s00122-019-03322-3 30879097 PMC6531409

[B9] Dreher K. Callis J. (2007). Ubiquitin, hormones and biotic stress in plants. *Ann. Bot.* 99 787–822. 10.1093/aob/mcl255 17220175 PMC2802907

[B10] Du X. Huang G. He S. Yang Z. Sun G. Ma X. (2018). Resequencing of 243 diploid cotton accessions based on an updated A genome identifies the genetic basis of key agronomic traits. *Nat. Genet.* 50 796–802. 10.1038/s41588-018-0116-x 29736014

[B11] Hao X. Tang H. Wang B. Yue C. Wang L. Zeng J. (2018). Integrative transcriptional and metabolic analyses provide insights into cold spell response mechanisms in young shoots of the tea plant. *Tree Physiol.* 38 1655–1671. 29688561 10.1093/treephys/tpy038

[B12] Hu Z. Deng G. Mou H. Xu Y. Chen L. Yang J. (2017). A re-sequencing-based ultra-dense genetic map reveals a gummy stem blight resistance-associated gene in *Cucumis melo*. *DNA Res.* 25 1–10. 10.1093/dnares/dsx033 28985339 PMC5824858

[B13] Huang X. Lu T. Han B. (2013). Resequencing rice genomes: an emerging new era of rice genomics. *Trends Genet.* 29 225–232. 10.1016/j.tig.2012.12.001 23295340

[B14] Jin J. Q. Chai Y. F. Liu Y. F. Zhang J. Yao M. Z. Chen L. (2018). Hongyacha, a naturally caffeine-free tea plant from Fujian, China. *J. Agric. Food Chem.* 66 11311–11319. 10.1021/acs.jafc.8b03433 30303011

[B15] Jin J. Q. Yao M. Z. Ma C. L. Ma J. Q. Chen L. (2016). Natural allelic variations of TCS1 play a crucial role in caffeine biosynthesis of tea plant and its related species. *Plant Physiol. Biochem.* 100 18–26. 10.1016/j.plaphy.2015.12.020 26773541

[B16] Lam H. M. Xu X. Liu X. Chen W. Yang G. Wong F. L. (2010). Resequencing of 31 wild and cultivated soybean genomes identifies patterns of genetic diversity and selection. *Nat. Genet.* 42 1053–1059. 10.1038/ng.715 21076406

[B17] Li X. Wu L. Wang J. Sun J. Xia X. Geng X. (2018). Genome sequencing of rice subspecies and genetic analysis of recombinant lines reveals regional yield- and quality-associated loci. *BMC Biol.* 16:102. 10.1186/s12915-018-0572-x 30227868 PMC6145349

[B18] Li Y. Wang X. Ban Q. Zhu X. Jiang C. Wei C. (2019). Comparative transcriptomic analysis reveals gene expression associated with cold adaptation in the tea plant *Camellia sinensis*. *BMC Genomics* 20:624. 10.1186/s12864-019-5988-3 31366321 PMC6670155

[B19] Liu S. An Y. Li F. Li S. Liu L. Zhou Q. (2018). Genome-wide identification of simple sequence repeats and development of polymorphic SSR markers for genetic studies in tea plant (*Camellia sinensis*). *Mol. Breed.* 38:59.

[B20] Liu S. An Y. Tong W. Qin X. Samarina L. Guo R. (2019). Characterization of genome-wide genetic variations between two varieties of tea plant (*Camellia sinensis*) and development of InDel markers for genetic research. *BMC Genomics* 20:935. 10.1186/s12864-019-6347-0 31805860 PMC6896268

[B21] Liu S. Liu H. Wu A. Hou Y. An Y. Wei C. (2017). Construction of fingerprinting for tea plant (*Camellia sinensis*) accessions using new genomic SSR markers. *Mol. Breed.* 37:93.

[B22] Liu Z. Zhu H. Liu Y. Kuang J. Zhou K. Liang F. (2016). Construction of a high-density, high-quality genetic map of cultivated lotus (*Nelumbo nucifera*) using next-generation sequencing. *BMC Genomics* 17:466. 10.1186/s12864-016-2781-4 27317430 PMC4912719

[B23] Meegahakumbura M. K. Wambulwa M. C. Li M. M. Thapa K. K. Sun Y. S. Moller M. (2017). Domestication origin and breeding history of the tea plant (*Camellia sinensis*) in China and India based on nuclear microsatellites and cpDNA Sequence Data. *Front. Plant Sci.* 8:2270. 10.3389/fpls.2017.02270 29422908 PMC5788969

[B24] Meegahakumbura M. K. Wambulwa M. C. Thapa K. K. Li M. M. Moller M. Xu J. C. (2016). Indications for three independent domestication events for the tea plant (*Camellia sinensis* (L.) O. Kuntze) and new insights into the origin of tea Germplasm in China and India revealed by nuclear microsatellites. *PLoS One* 11:e0155369. 10.1371/journal.pone.0155369 27218820 PMC4878758

[B25] Niu S. Song Q. Koiwa H. Qiao D. Zhao D. Chen Z. (2019). Genetic diversity, linkage disequilibrium, and population structure analysis of the tea plant (*Camellia sinensis*) from an origin center, Guizhou plateau, using genome-wide SNPs developed by genotyping-by-sequencing. *BMC Plant Biol.* 19:328. 10.1186/s12870-019-1917-5 31337341 PMC6652003

[B26] Notaguchi M. Wolf S. Lucas W. J. (2012). Phloem-mobile Aux/IAA transcripts target to the root tip and modify root architecture. *J Integr Plant Biol.* 54 760–772. 10.1111/j.1744-7909.2012.01155.x 22925478

[B27] Ogutu C. Cherono S. Ntini C. Mollah M. D. Zhao L. Belal M. A. (2020). Evolutionary rate variation among genes involved in galactomannan biosynthesis in Coffea canephora. *Ecol. Evol.* 10 2559–2569. 10.1002/ece3.6084 32185001 PMC7069334

[B28] Ploense S. E. Wu M. F. Nagpal P. Reed J. W. (2009). A gain-of-function mutation in IAA18 alters *Arabidopsis* embryonic apical patterning. *Development* 136 1509–1517. 10.1242/dev.025932 19363152 PMC2674258

[B29] Qian C. Chen Z. Liu Q. Mao W. Chen Y. Tian W. (2020). Coordinated transcriptional regulation by the UV-B photoreceptor and multiple transcription factors for plant UV-B responses. *Mol. Plant* 13 777–792. 10.1016/j.molp.2020.02.015 32126287

[B30] Saxena R. K. Edwards D. Varshney R. K. (2014). Structural variations in plant genomes. *Brief. Funct. Genomics* 13 296–307. 10.1093/bfgp/elu016 24907366 PMC4110416

[B31] Shang Y. Ma Y. Zhou Y. Zhang H. Duan L. Chen H. (2014). Plant science. Biosynthesis, regulation, and domestication of bitterness in cucumber. *Science* 346 1084–1088. 10.1126/science.1259215 25430763

[B32] Shen C. Jin X. Zhu D. Lin Z. (2017). Uncovering SNP and indel variations of tetraploid cottons by SLAF-seq. *BMC Genomics* 18:247. 10.1186/s12864-017-3643-4 28330454 PMC5363057

[B33] Wang H. Jones B. Li Z. Frasse P. Delalande C. Regad F. (2005). The tomato Aux/IAA transcription factor IAA9 is involved in fruit development and leaf morphogenesis. *Plant Cell* 17 2676–2692. 10.1105/tpc.105.033415 16126837 PMC1242265

[B34] Wang H. Xu X. Vieira F. G. Xiao Y. Li Z. Wang J. (2016). The power of inbreeding: NGS-Based GWAS of rice reveals convergent evolution during rice domestication. *Mol. Plant* 9 975–985. 10.1016/j.molp.2016.04.018 27179918

[B35] Wang M. Tu L. Lin M. Lin Z. Wang P. Yang Q. (2017). Asymmetric subgenome selection and cis-regulatory divergence during cotton domestication. *Nat. Genetics* 49 579–587. 10.1038/ng.3807 28263319

[B36] Wang N. Zhang Y. Huang S. Liu Z. Li C. Feng H. (2020). Defect in Brnym1, a magnesium-dechelatase protein, causes a stay-green phenotype in an EMS-mutagenized Chinese cabbage (*Brassica campestris* L. ssp. *pekinensis*) line. *Hortic. Res.* 7:8. 10.1038/s41438-019-0223-6 PMC6944686 31934339

[B37] Wei C. Yang H. Wang S. Zhao J. Liu C. Gao L. (2018). Draft genome sequence of *Camellia sinensis* var. *sinensis* provides insights into the evolution of the tea genome and tea quality. *Proc. Natl. Acad. Sci. U. S. A.* 115 E4151–E4158. 29678829 10.1073/pnas.1719622115 PMC5939082

[B38] Wu J. Wang Y. Xu J. Korban S. S. Fei Z. Tao S. (2018). Diversification and independent domestication of Asian and European pears. *Genome Biol.* 19:77. 10.1186/s13059-018-1452-y PMC5996476 29890997

[B39] Xanthopoulou A. Montero-Pau J. Mellidou I. Kissoudis C. Blanca J. Pico B. (2019). Whole-genome resequencing of *Cucurbita* pepo morphotypes to discover genomic variants associated with morphology and horticulturally valuable traits. *Hortic. Res.* 6:94. 10.1038/s41438-019-0176-9 PMC6804688 31645952

[B40] Xia E. Tong W. Hou Y. An Y. Chen L. Wu Q. (2020a). The reference genome of tea plant and resequencing of 81 diverse accessions provide insights into genome evolution and adaptation of tea plants. *Mol. Plant* 13 1013–1026. 10.1016/j.molp.2020.04.010 32353625

[B41] Xia E. H. Tong W. Wu Q. Wei S. Zhao J. Zhang Z. Z. (2020b). Tea plant genomics: achievements, challenges and perspectives. *Hortic. Res.* 7:7. 10.1038/s41438-019-0225-4 PMC6938499 31908810

[B42] Xia E. H. Zhang H. B. Sheng J. Li K. Zhang Q. J. Kim C. (2017). The tea tree genome provides insights into tea flavor and independent evolution of caffeine biosynthesis. *Mol. Plant* 10 866–877. 10.1016/j.molp.2017.04.002 28473262

[B43] Yang H. Wei C. L. Liu H. W. Wu J. L. Li Z. G. Zhang L. (2016). Genetic Divergence between *Camellia sinensis* and Its Wild Relatives Revealed via Genome-Wide SNPs from RAD Sequencing. *PLoS One* 11:e0151424. 10.1371/journal.pone.0151424 26962860 PMC4786323

[B44] Yang Z. Yoder A. D. (1999). Estimation of the transition/transversion rate bias and species sampling. *J. Mol. Evol* 48 274–283. 10.1007/pl00006470 10093216

[B45] Zhang Q. Zhang H. Sun L. Fan G. Ye M. Jiang L. (2018). The genetic architecture of floral traits in the woody plant Prunus mume. *Nat. Commun.* 9:1702. 10.1038/s41467-018-04093-z PMC5923208 29703940

[B46] Zhang T. Gu M. Liu Y. Lv Y. Zhou L. Lu H. (2017). Development of novel InDel markers and genetic diversity in *Chenopodium quinoa* through whole-genome re-sequencing. *BMC Genomics* 18:685. 10.1186/s12864-017-4093-8 28870149 PMC5584319

[B47] Zhao D.-W. Yang J.-B. Yang S.-X. Kato K. Luo J.-P. (2014a). Genetic diversity and domestication origin of tea plant *Camellia taliensis* (Theaceae) as revealed by microsatellite markers. *BMC Plant Biol.* 14:14. 10.1016/j.pld.2020.06.003 24405939 PMC3890520

[B48] Zhao Y. Chan Z. Gao J. Xing L. Cao M. Yu C. (2016). ABA receptor PYL9 promotes drought resistance and leaf senescence. *Proc. Natl. Acad. Sci. U. S. A.* 113 1949–1954. 10.1073/pnas.1522840113 26831097 PMC4763734

[B49] Zhao Y. Xing L. Wang X. Hou Y. J. Gao J. Wang P. (2014b). The ABA receptor PYL8 promotes lateral root growth by enhancing MYB77-dependent transcription of auxin-responsive genes. *Sci. Signal* 7:ra53. 10.1126/scisignal.2005051 24894996 PMC4298826

[B50] Zheng C. Wang Y. Ding Z. Zhao L. (2016). Global transcriptional analysis reveals the complex relationship between tea quality, leaf senescence and the responses to cold-drought combined stress in *Camellia sinensis*. *Front. Plant Sci.* 7:1858. 10.3389/fpls.2016.01858 28018394 PMC5145883

[B51] Zhu B. Chen L. B. Lu M. Zhang J. Han J. Deng W. W. (2019). Caffeine content and related gene expression: novel insight into caffeine metabolism in *Camellia* plants containing low, normal, and high caffeine concentrations. *J. Agric. Food Chem.* 67 3400–3411. 10.1021/acs.jafc.9b00240 30830771

[B52] Zsogon A. Cermak T. Naves E. R. Notini M. M. Edel K. H. Weinl S. (2018). *De novo* domestication of wild tomato using genome editing. *Nat. Biotechnol.* 36 1211–1216. 10.1038/nbt.4272 30272678

