# Supplementary material for: Revealing Distinctions in Genetic Diversity and Adaptive Evolution Between Two Varieties of Camellia sinensis by Whole-Genome Resequencing
Source: Front Plant Sci. 2020 Nov 24;11:603819. doi: 10.3389/fpls.2020.603819 (PMC7732639; doi:10.3389/fpls.2020.603819)
Supplement: Supplementary file 1 [file Table_1.pdf]

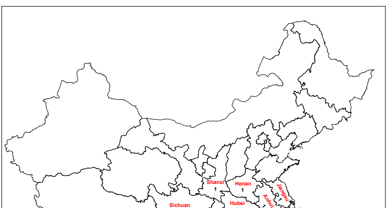

FIGURE. S1 | The geographical distribution of 33 sequenced materials. The numbers in the map represent the number of samples from different provinces.

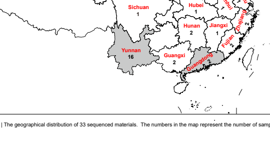

FIGURE. S2 | KEGG functional categorization of the genes contained in differentiation region.

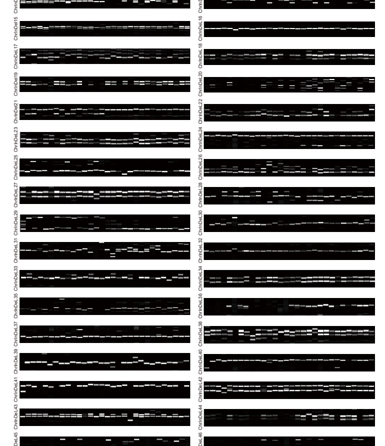

FIGURE. S3 | Exhibition of transferability and polymorphism detected of 53 indel markers among 30 tea varieties.

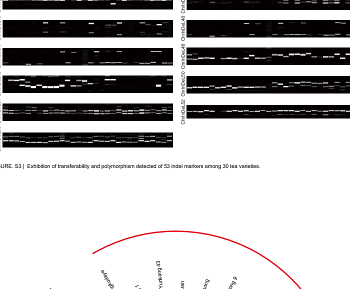

FIGURE. S4 | Dendrogram constructed based on genotypes from the 53 indel markers using neighbor-joining algorithm with 1000 bootstrap replicates.

TABLE S1 | Summary of the sequencing data of collected tea accessions.

Accession name	Clean_Reads	Clean_Base	Q20(%)	Q30(%)	GC(%)	Cov_ratio_1X(%)	Cov_ratio_5X(%)	Properly_mapped(%)
Anhui 3	153817249	4.6065E+10	96.43	90.98	38.7	90.04	76.12	86.42
Fudingdabai	176512128	5.2856E+10	96.14	90.43	38.79	93.11	79.52	87.32
Longjing 43	159289183	4.769E+10	96.5	91.07	38.55	89.88	76.8	87.44
Baihaozao	188219934	5.6348E+10	96.58	91.07	39.16	90.11	78.51	87.26
Xinyang 10	152822669	4.5775E+10	95.92	90.08	38.52	90.39	76.58	85.2
Guihong 3	157828048	4.728E+10	95.97	90.15	38.75	90.27	76.76	86.23
Echa 5	182600554	5.4703E+10	97.23	92.79	38.85	91.42	80.11	86.81
Chuanmu 28	164916404	4.9393E+10	97.19	92.64	38.5	90.08	76.61	86.88
Xicha 5	188870332	5.658E+10	97.27	92.87	38.55	90.7	79.71	86.24
Gancha 3	187679528	5.6215E+10	96.94	92.11	38.66	91.24	80.08	86.82
Qucha	154732683	4.6354E+10	97.24	92.71	37.96	88.88	75.09	86.48
Shancha 1	205834482	6.1665E+10	97.1	92.47	38.76	91.11	80.78	85.97
Xiaoxianghong 21-3	190890724	5.7185E+10	97.2	92.72	38.8	91.53	79.95	85.74
Zhenong 117	165503449	4.9578E+10	96.16	90.2	38.99	90.6	77.03	85.4
Yaoshanxiulv	163302278	4.8912E+10	96.95	92.14	38.77	90.05	77.2	85.36
Yunkang 10	151448823	4.5362E+10	95.95	90	38.76	91.11	75.32	85.8
Zijuan	167863321	5.0284E+10	97	92.31	38.66	91.25	77	84.98
Xiuhong	136101997	4.0931E+10	96.44	90.71	40.3	90	70.97	86.12
Yinghong 9	154044314	4.6303E+10	96.57	91.06	39.54	89.93	70.43	84.86
Qingshui 3	152272439	4.5778E+10	96.52	90.92	40.63	89.93	72.64	86.64
Changyebaihao	144738226	4.351E+10	96.61	91	39.57	85.83	67.16	84.67
Yunkang 43	150712537	4.5314E+10	96.5	90.89	39.54	88.32	71.28	85.65
Yunxuan 9	166628697	5.0101E+10	96.62	91.16	39.69	86.12	69.64	83.87
Shuangjiangheidaye	158280548	4.7506E+10	96.67	91.44	39.97	86	68.67	83.77
Menghaidaye	156643707	4.7062E+10	96.63	91.23	40.05	86.83	68.95	84.54
Mengkudaye	168322456	5.0595E+10	96.57	91.01	39.89	93.02	75.9	85.15
Yunkang 14	146670895	4.4033E+10	96.48	90.99	39.83	88.65	70.56	84.07
Yunkang 37	149547041	4.499E+10	96.36	90.68	39.63	88.64	71.43	85.2
Nannuoshan 14	185189034	5.5671E+10	96.59	91.12	40.19	87.44	71.79	79.36
Hekai 33	153108601	4.6038E+10	96.58	91.04	39.77	85.68	67.59	82.61
CTS-1	151312511	4.5532E+10	96.31	90.56	40.29	79.99	59.2	78.31
CTS-2	173381909	5.2114E+10	96.6	91.12	41.35	79.38	59.12	76.46
CTS-3	163392044	4.9125E+10	96.63	91.08	41.02	79.38	59.42	76.8

TABLE S2 | Selective sweep regions identified by Fst and θπ between CSS and CSA population.

Selective sweep genomic regions						Genes within selective sweep regions	
CSS			CSA			CSS	CSA
Chromosome	Start	End	Chromosome	Start	End	GeneID	GeneID
CHR2	68050001	68550000	CHR1	10200001	10700000	CSS0000114.1	CSS0000184.2
CHR2	68100001	68600000	CHR1	10250001	10750000	CSS0000133.1	CSS0000788.1
CHR2	68150001	68650000	CHR2	26800001	27300000	CSS0000451.1	CSS0001385.1
CHR2	68200001	68700000	CHR2	177250001	177750000	CSS0000509.1	CSS0001424.1
CHR2	68250001	68750000	CHR2	177300001	177800000	CSS0000564.1	CSS0001703.1
CHR2	85350001	85850000	CHR2	177350001	177850000	CSS0000783.1	CSS0001801.1
CHR2	87700001	88200000	CHR2	177400001	177900000	CSS0000899.1	CSS0002695.1
CHR2	96550001	97050000	CHR2	177450001	177950000	CSS0000919.1	CSS0003083.1
CHR2	96600001	97100000	CHR2	177500001	178000000	CSS0000963.1	CSS0003137.1
CHR2	96650001	97150000	CHR2	177550001	178050000	CSS0000972.1	CSS0004427.1
CHR2	96700001	97200000	CHR2	178950001	179450000	CSS0001124.1	CSS0004627.1
CHR2	96750001	97250000	CHR2	179000001	179500000	CSS0001125.1	CSS0005470.1
CHR2	96800001	97300000	CHR4	41150001	41650000	CSS0001139.1	CSS0005472.1
CHR2	96850001	97350000	CHR4	41200001	41700000	CSS0001694.1	CSS0005860.1
CHR2	96900001	97400000	CHR4	52150001	52650000	CSS0001700.1	CSS0005914.1
CHR2	96950001	97450000	CHR4	52200001	52700000	CSS0001715.1	CSS0006441.1
CHR2	97000001	97500000	CHR4	52250001	52750000	CSS0001863.1	CSS0006712.1
CHR2	97050001	97550000	CHR4	52300001	52800000	CSS0002328.1	CSS0006929.1
CHR2	143550001	144050000	CHR4	52350001	52850000	CSS0002377.1	CSS0006937.1
CHR2	143600001	144100000	CHR5	14100001	14600000	CSS0002632.1	CSS0007619.1
CHR2	143650001	144150000	CHR5	14150001	14650000	CSS0002670.1	CSS0008568.1
CHR2	143700001	144200000	CHR5	14200001	14700000	CSS0002685.1	CSS0008695.1
CHR2	143750001	144250000	CHR5	14250001	14750000	CSS0002991.1	CSS0009003.1
CHR2	143800001	144300000	CHR5	19100001	19600000	CSS0003172.1	CSS0009052.1
CHR2	155350001	155850000	CHR5	19150001	19650000	CSS0003214.1	CSS0009076.1
CHR2	155400001	155900000	CHR6	141250001	141750000	CSS0003220.1	CSS0009432.1
CHR2	155450001	155950000	CHR6	141300001	141800000	CSS0003240.1	CSS0009931.1
CHR2	155500001	156000000	CHR7	142800001	143300000	CSS0003257.1	CSS0010716.1
CHR2	155550001	156050000	CHR7	154800001	155300000	CSS0003266.2	CSS0010812.1
CHR2	155600001	156100000	CHR7	154850001	155350000	CSS0003420.1	CSS0011069.1
CHR2	155650001	156150000	CHR7	154900001	155400000	CSS0003657.1	CSS0011195.1
CHR2	165100001	165600000	CHR7	175750001	176250000	CSS0003717.1	CSS0011345.2
CHR2	166550001	167050000	CHR7	175800001	176300000	CSS0003755.1	CSS0014155.1
CHR2	166600001	167100000	CHR7	175850001	176350000	CSS0003769.1	CSS0014477.1
[illegible]	[illegible]	[illegible]	[illegible]	[illegible]	[illegible]	[illegible]	[illegible]

TABLE S3 | Primer information of indel marker newly developed in this study.

Marker ID	Forward primer (5'-3')	Reverse primer(5'-3')
ChrInDel01	AATTCACCTCCAATGTCCACC	TGCTCTTTCAGTTGGCAGTAA
ChrInDel02	GATCGAGTCATTTGTGGTGGA	ACGAGCAGAGGACTATGTACA
ChrInDel03	CCCACAAGAGTCTACAGAGGA	GCTGGTTTAAAATGCAGCCAT
ChrInDel04	GAACTTGCAGGTGTAGAAGGA	GGTCATCCCAAACAACCCTTA
ChrInDel05	GTTTTCCTGCACTTGGTTAGC	ATTGCCTTGTACAGATGGGAC
ChrInDel06	AAACTCACATCATTCCCCCTC	TGGGTTGGGTAATGAAGCTTT
ChrInDel07	GAGTGGATTAGGAGGGCTTTG	TGAGGACCTGGGATTCTAGTT
ChrInDel08	ATGGAGCTGGGTCTGATTCTA	ACAGTTCCGTTTTCTAGTGGA
ChrInDel09	AAACCTCTACCAAACGCTACC	TGTCCAACAAACCAAACCAT
ChrInDel10	TGGGTTTGGGTCTTTGATCTG	TTACAACTTGAGTGGCCAACA
ChrInDel11	GGCCCTATTACGAGTGAGTTC	TTAGGGACGTGCTCTTTCATG
ChrInDel12	CTCCTTCCTCTCTCCCTCTTT	ATTGTGCAGCTGAAGGAGTAG
ChrInDel13	TGGGACAGAGAAAGAGCAAAG	TTGTGTGAAAGCCCAACTACA
ChrInDel14	GGGATGGTTTTGAAGCGAAAG	AATCTACGCTACACAACCCAC
ChrInDel15	TTTAGCTAATCACCCCACTGC	ACTCGCTTACAACTCGTCAAA
ChrInDel16	ATTGGATGTTTGAGCCCATGA	AATCATCGCCGAAACAAATGG
ChrInDel17	GCATCACCAATACTATTAGGCA	GTCTCAACTAACATTTACTGGC
ChrInDel18	AACAATGGCCAGAAGTCATGT	TCCCTCCATTCGGTAAGAGAA
ChrInDel19	GAGTTCAGGCCAATGGTAGAG	TTACCGCAACCGTTACACTAC
ChrInDel20	AGCAAACTGGACGCATAGAAT	AACTTGGACATGAGGAGTTCG
ChrInDel21	TGGAGCTGAAATTTGTGTCCT	TGAGCAAATCTTAGTGGGTGG
ChrInDel22	TCCCCTTCATGTTCGAGTTTT	GGTAACCTCGGCATAACTACC
ChrInDel23	TAGTCTTTGCCGAACTCCAAG	GAGCAAGCCAGAAATCTCAGA
ChrInDel24	AGATGCTATTGTTTCCCTGCT	GGGTTCATGTTTAGGTTTGCG
ChrInDel25	GTCAAACGGGATTGTCAATAAC	GAAGTACCGTACCATGCACAG
ChrInDel26	TCAGCCACAGATCTTTGAAGG	ACCTCTGTCCAGTCCAGTAAA
ChrInDel27	GCTACACAACAACATCTACAGC	GGTTAATGCTGAGCTACACCA
ChrInDel28	GCCTAAATGTTGTCCAGACCA	GAGTCTTCTTGGAGCGAATGT
ChrInDel29	TGTTTGTCTGTGTGTGTTTGG	TCAGACAAAAGAAGTGCAGCT
ChrInDel30	AGGAAACAAGAAGTGGGGAAA	AAACCTATCCAACACGAGCTG
ChrInDel31	AATGGTTTGCTGCAGAACTTG	TCTCCCACCCCTTCAAAAATG
ChrInDel32	GAGTACCCTTATTTGCTTTCGC	TGGAGTGAGTATTGCATTGGG
ChrInDel33	GCCAAACAAGCTCAAATCACT	TTATTTTGTTGGGAAAGCGCC
ChrInDel34	GCCAAACAAGCTCAAATCACT	AATCAACCCACATGCCATCTG
ChrInDel35	AACGCATCGCAAATACAACCTA	AGAACTGTGCAAAGTATGGCT
ChrInDel36	TCTTGTGAAGGCCTTTGTTGT	AAGGTGCTGTGAAATCGATCA
ChrInDel37	GTGTAGGGTTTCATCTCTGGC	ACAGGGACCAAATTGACACAT
ChrInDel38	AAGCTGCAAATCCTGATCAGT	ACAGCATACGACACTTTCTCC
ChrInDel39	AACTACTGAAGACCTCCACCA	TGTCTTTCCAGATTGCCCAAA
ChrInDel40	CCAGGTCAGAAAAACGATGGA	AGCTCCACCAACCAATTCTAC
ChrInDel41	TGTGCTGATTTGACTTGTTGG	AGTACCTATAACACCCAGCGA
ChrInDel42	TGTAATGGAGGAGAGAGGGAC	GTCCAATCAAACGCACCATTT
ChrInDel43	ACTTTTTGGTATGAGCTGTTGG	AGGAGCATGTGTGGATAAGAC
ChrInDel44	GCTTTAGTCAGGGGTATAGATG	TGAGGGCATCTAGTGAACAGT
ChrInDel45	TGTAATGCGCACGTTAAACAC	TGAGGGCATCTAGTGAACAGT
ChrInDel46	GCCCAACAGTAACCTTCAAT	TCTATACCCCTGACTAAAGCT
ChrInDel47	ACCCTTGCCACCATTTTAGTT	GTTTGTGATGTGTCTAATGTGC
ChrInDel48	TGAGGGATAAAATTGGAGCATC	GCATGATCACGAGGCCTTAAT
ChrInDel49	TGAGGGATAAAATTGGAGCATC	GTACGTGCGATTAAGGATGGA
ChrInDel50	TCACCATCATTGTCGTCATCA	GGAGATAGTGGTGGTAGTGGA
ChrInDel51	TCACCATCATTGTCGTCATCA	GCCTATGCGAAAGAGTTTGTG
ChrInDel52	GGTCACTGCTGCATCTACAAA	GGCCCAACTAGACTTGTCATT
ChrInDel53	CGAAGCCCAACAGATTTCAAG	ACTCGAGCACATTAGTTTTCCA

TABLE S4 | Detailed information of 33 tea tree samples used for resequencing.

Sample ID	English name	Subspecies	Germplasm type	Registration number in China	Provinces	Sample code in NCBI
1	'Anhui 3'	*C. sinensis* var. *sinensis*	NTC	GS13009-1987	Anhui	SAMN14785849
2	'Fudingdabai'	*C. sinensis* var. *sinensis*	NTC	GS13001-1985	Fujian	SAMN14785852
3	'Longjing 43'	*C. sinensis* var. *sinensis*	NTC	GS13007-1987	Zhejiang	SAMN14785854
4	'Baihaozao'	*C. sinensis* var. *sinensis*	NTC	GS13017-1994	Hunan	SAMN14785858
5	'Xinyang 10'	*C. sinensis* var. *sinensis*	NTC	GS13011-1994	Henan	SAMN14785860
6	'Guihong 3'	*C. sinensis* var. *sinensis*	NTC	GS13001-1994	Guangxi	SAMN14785863
7	'Echa 5'	*C. sinensis* var. *sinensis*	NTC	GS2010018	Hubei	SAMN14785864
8	'Chuanmu 28'	*C. sinensis* var. *sinensis*	PTC	-	Sichuan	SAMN14785874
9	'Xicha 5'	*C. sinensis* var. *sinensis*	NTC	GS13005-1994	Jiangsu	SAMN14785879
10	'Gancha 3'	*C. sinensis* var. *sinensis*	PTC	-	Jiangxi	SAMN14785881
11	'Qucha'	*C. sinensis* var. *sinensis*	LTC	-	Fujian	SAMN14785871
12	'Shancha 1'	*C. sinensis* var. *sinensis*	NTC	GP610009	Shanxi	SAMN14785865
13	'Xiaoxianghong 21-3'	*C. sinensis* var. *sinensis*	PTC	XPD008-2012	Hunan	SAMN14785872
14	'Zhenong 117'	*C. sinensis* var. *sinensis*	PTC	2006001	Zhejiang	SAMN14785884
15	'Yaoshanxiulv'	*C. sinensis* var. *sinensis*	NTC	GS2010008	Guangxi	SAMN14785885
16	'Yunkang 10'	*C. sinensis* var. *assamica*	PTC	GS13020-1987	Yunnan	SAMN14785861
17	'Zijuan'	*C. sinensis* var. *assamica*	LTC	-	Yunnan	SAMN14785873
18	'Xiuhong'	*C. sinensis* var. *assamica*	NTC	GS2002003	Guangdong	SAMN14785918
19	'Yinghong 9'	*C. sinensis* var. *assamica*	PTC	-	Guangdong	SAMN14785919
20	'Qingshui 3'	*C. sinensis* var. *assamica*	LTC	-	Yunnan	SAMN14785890
21	'Changyebaihao'	*C. sinensis* var. *assamica*	PTC	-	Yunnan	SAMN14785891
22	'Yunakng 43'	*C. sinensis* var. *assamica*	PTC	-	Yunnan	SAMN14785892
23	'Yunxuan 9'	*C. sinensis* var. *assamica*	PTC	-	Yunnan	SAMN14785893
24	'Shuangjiangdaheiye'	*C. sinensis* var. *assamica*	LTC	-	Yunnan	SAMN14785895
25	'Menghaidaye'	*C. sinensis* var. *assamica*	NTC	-	Yunnan	SAMN14785896
26	'Mengkudaye'	*C. sinensis* var. *assamica*	NTC	GS13012-1985	Yunnan	SAMN14785897
27	'Yunkang 14'	*C. sinensis* var. *assamica*	NTC	GS13021-1987	Yunnan	SAMN14785898
28	'Yunkang 37'	*C. sinensis* var. *assamica*	PTC	-	Yunnan	SAMN14785899
29	'Nannuoshan 14'	*C. sinensis* var. *assamica*	LTC	-	Yunnan	SAMN14785900
30	'Hekai 33'	*C. sinensis* var. *assamica*	LTC	-	Yunnan	SAMN14785903
31	'CTS-1'	*Camellia taliensis*	Wild	-	Yunnan	SAMN14785905
32	'CTS-2'	*Camellia taliensis*	Wild	-	Yunnan	SAMN14785906
33	'CTS-3'	*Camellia taliensis*	Wild	-	Yunnan	SAMN14785907

Note: NTC, PTC and LTC represent national tea varieties, provincial tea varieties and local tea varieties respectively.

TABLE S5 | Details information of 30 tea tree samples used for indel markers verification.

Sample ID	English name	Subspecies	Germplasm type	Registration number in China	Provinces
1	'Fudingdabai'	*C. sinensis* var. *sinensis*	NTC	GS13001-1985	Fujian
2	'Baihaozao'	*C. sinensis* var. *sinensis*	NTC	GS13017-1994	Hunan
3	'Shancha 1'	*C. sinensis* var. *sinensis*	NTC	GP610009	Shanxi
4	'Zijuan'	*C. sinensis* var. *assamica*	LTC	-	Yunnan
5	'Xiuhong'	*C. sinensis* var. *assamica*	NTC	GS2002003	Guangdong
6	'Yinghong 9'	*C. sinensis* var. *assamica*	PTC	-	Guangdong
7	'Qingshui 3'	*C. sinensis* var. *assamica*	LTC	-	Yunnan
8	'Changyebaihao'	*C. sinensis* var. *assamica*	PTC	-	Yunnan
9	'Yunakng 43'	*C. sinensis* var. *assamica*	PTC	-	Yunnan
10	'Yunxuan 9'	*C. sinensis* var. *assamica*	PTC	-	Yunnan
11	'Shuangjiangdaheiye'	*C. sinensis* var. *assamica*	LTC	-	Yunnan
12	'Menghaidaye'	*C. sinensis* var. *assamica*	NTC	-	Yunnan
13	'Mengkudaye'	*C. sinensis* var. *assamica*	NTC	GS13012-1985	Yunnan
14	'Yunkang 14'	*C. sinensis* var. *assamica*	NTC	GS13021-1987	Yunnan
15	'Yunkang 37'	*C. sinensis* var. *assamica*	PTC	-	Yunnan
16	'Nannuoshan 14'	*C. sinensis* var. *assamica*	LTC	-	Yunnan
17	'Hekai 33'	*C. sinensis* var. *assamica*	LTC	-	Yunnan
18	'Shuchazao'	*C. sinensis* var. *sinensis*	NTC	GS2002008	Anhui
19	'Rougui'	*C. sinensis* var. *sinensis*	PTC	1985002	Fujian
20	'Echa 1'	*C. sinensis* var. *sinensis*	NTC	GS2002013	Hubei
21	'Zhongcha 108'	*C. sinensis* var. *sinensis*	NTC	GS2010013	Zhejiang
22	'Longjingchangye'	*C. sinensis* var. *sinensis*	NTC	GS13008-1994	Zhejiang
23	'Hongyan 12'	*C. sinensis* var. *sinensis*	NTC	GS2010020	Guangdong
24	'Zhenong 113'	*C. sinensis* var. *sinensis*	NTC	GS13009-1994	Zhejiang
25	'Fenghuangshuixian'	*C. sinensis* var. *sinensis*	NTC	GS13017-1985	Guangdong
26	'Tieguanyin'	*C. sinensis* var. *sinensis*	NTC	GS13007-1985	Fujian
27	'Huangshanbaicha 3'	*C. sinensis* var. *sinensis*	NTC	-	Anhui
28	'Guihong 1'	*C. sinensis* var. *sinensis*	LTC	-	Guangxi
29	'Qiammei 601'	*C. sinensis* var. *assamica*	NTC	GS13013-1994	Guizhou
30	'Foxiang 1'	*C. sinensis* var. *assamica*	PTC	DS027-2003	Yunnan

Note: NTC, PTC and LTC represent national tea varieties, provincial tea varieties and local tea varieties respectively.
The samples marked in red represent the same samples as in TABLE S4.
